# Contributions of diseases and injuries to widening life expectancy inequalities in England from 2001 to 2016: a population-based analysis of vital registration data

**DOI:** 10.1016/S2468-2667(18)30214-7

**Published:** 2018-11-23

**Authors:** James E Bennett, Jonathan Pearson-Stuttard, Vasilis Kontis, Simon Capewell, Ingrid Wolfe, Majid Ezzati

**Affiliations:** a Department of Epidemiology and Biostatistics, School of Public Health, Imperial College London, London, UK; b MRC-PHE Centre for Environment and Health, Imperial College London, London, UK; c WHO Collaborating Centre on NCD Surveillance and Epidemiology, Imperial College London, London, UK; d Department of Public Health and Policy, University of Liverpool, Liverpool, UK; e Department of Primary Care and Public Health Sciences, King's College London, London, UK; f Evelina London Children's Healthcare, Guy's and St Thomas' NHS Trust, London, UK

## Abstract

**Background:**

Life expectancy inequalities in England have increased steadily since the 1980s. Our aim was to investigate how much deaths from different diseases and injuries and at different ages have contributed to this rise to inform policies that aim to reduce health inequalities.

**Methods:**

We used vital registration data from the Office for National Statistics on population and deaths in England, by underlying cause of death, from 2001 to 2016, stratified by sex, 5-year age group, and decile of the Index of Multiple Deprivation (based on the ranked scores of Lower Super Output Areas in England in 2015). We grouped the 7·65 million deaths by their assigned International Classification of Diseases (10th revision) codes to create categories of public health and clinical relevance. We used a Bayesian hierarchical model to obtain robust estimates of cause-specific death rates by sex, age group, year, and deprivation decile. We calculated life expectancy at birth by decile of deprivation and year using life-table methods. We calculated the contributions of deaths from each disease and injury, in each 5-year age group, to the life expectancy gap between the most deprived and affluent deciles using Arriaga's method.

**Findings:**

The life expectancy gap between the most affluent and most deprived deciles increased from 6·1 years (95% credible interval 5·9–6·2) in 2001 to 7·9 years (7·7–8·1) in 2016 in females and from 9·0 years (8·8–9·2) to 9·7 years (9·6–9·9) in males. Since 2011, the rise in female life expectancy has stalled in the third, fourth, and fifth most deprived deciles and has reversed in the two most deprived deciles, declining by 0·24 years (0·10–0·37) in the most deprived and 0·16 years (0·02–0·29) in the second-most deprived by 2016. Death rates from every disease and at every age were higher in deprived areas than in affluent ones in 2016. The largest contributors to life expectancy inequalities were deaths in children younger than 5 years (mostly neonatal deaths), respiratory diseases, ischaemic heart disease, and lung and digestive cancers in working ages, and dementias in older ages. From 2001 to 2016, the contributions to inequalities declined for deaths in children younger than 5 years, ischaemic heart disease (for both sexes), and stroke and intentional injuries (for men), but increased for most other causes.

**Interpretation:**

Recent trends in life expectancy in England have not only resulted in widened inequalities but the most deprived communities are now seeing no life expectancy gain. These inequalities are driven by a diverse group of diseases that can be effectively prevented and treated. Adoption of the principle of proportionate universalism to prevention and health and social care can postpone deaths into older ages for all communities and reduce life expectancy inequalities.

**Funding:**

Wellcome Trust.

## Introduction

Life expectancy inequality between affluent and deprived communities has increased steadily in England since the 1980s.^1^, ^2^ Health inequalities can be tackled through progressive economic and social policies; public health interventions that reduce inequalities in environmental, nutritional, and behavioural risks; and preventive and life-saving treatments to delay the onset of disease or death in disadvantaged groups.^3^

Some of these options, such as cancer screening, affect specific diseases. Others, such as policies that reduce poverty and social inequalities and investments in health and social care, influence the occurrence and outcomes of many diseases. Information on the contribution of deaths from different diseases and injuries, and at different ages, to life expectancy inequalities is needed to envision how each intervention and policy option affects not only aggregate health outcomes but also health inequalities. Studying how these contributions have changed over time can indicate which diseases and injuries are driving the widening health inequalities, and thereby helps policy makers and civil society to make specific recommendations for improving health.

Although life expectancy inequalities in England have been documented, there is little information on how much deaths from specific diseases and at different ages contribute to these inequalities^4^, ^5^ and how these contributions have changed. We used national statistics data on deaths by age group and underlying cause of death to estimate their respective contributions to life expectancy inequalities in England from 2001 to 2016.

**Research in context**

**Evidence before this study**

We searched PubMed for articles published from inception up to Aug 14, 2018, with search terms “life expectancy” AND “inequality” AND (“trend” OR “decomposition”) AND (“England” or “United Kingdom”) for papers that had analysed life expectancy inequalities in England or the UK and the contributions of deaths at different ages and from different diseases and injuries to these inequalities, with no language restrictions. We also searched for reports on health inequalities through the websites of the Office for National Statistics and Public Health England. We found several articles and reports on life expectancy inequalities in England at one point in time and two articles on trends in life expectancy inequalities. We found two consecutive annual reports that quantified separately the contributions of deaths at different ages and from different diseases and injuries to the life expectancy difference between the top and bottom deciles of deprivation at one point in time, but none that had done so over time nor that considered the combination of disease and age groups.

**Added value of this study**

To our knowledge, this Article shows for the first time how deaths at different ages and from different diseases contribute to the life expectancy inequality between deprived and affluent groups in England, and how these contributions have changed as the extent of inequality has increased. We used a Bayesian statistical model so that our estimates of life expectancy inequality, and of contributions of deaths by age group and disease or injury to these inequalities, were robust to small numbers of deaths in some age groups and diseases.

**Implications of all the available evidence**

Life expectancy inequality between deprived and affluent areas of England has increased since 2001. Currently, the main contributors to life expectancy inequality are deaths in children younger than 5 years, respiratory diseases, ischaemic heart disease, lung and digestive cancers in working ages, and dementias in older ages. Over time, the contributions of deaths in children younger than 5 years, ischaemic heart disease and stroke (for both sexes), and intentional injuries (for men) to inequalities have declined but those of most other disease groups have increased. The diversity of diseases that contribute to life expectancy inequalities demonstrates the need for policies that address poverty and social inequalities coupled with investment in the National Health Service to improve high-quality primary and specialist care for deprived groups and communities under the principle of proportionate universalism. Specific public health and clinical interventions—for example, fiscal and regulatory measures to reduce alcohol and tobacco use and technology for early diagnosis—might also help to reduce inequalities in life expectancy if targeted towards deprived communities and social groups.

## Methods

### Data sources

We obtained yearly data from the Office for National Statistics on population and all deaths, by underlying cause of death, from 2001 to 2016 in England. We started the analysis from 2001 because the change from the International Classification of Diseases (ICD), 9th revision, to ICD-10 in that year affects death counts for some causes of death, and hence restricts comparisons over time. The underlying causes of death were divided into categories on the basis of their assigned ICD-10 codes (appendix p 15). These categories were selected because of their public health and clinical relevance in terms of interventions and to ensure cause groups contained a sufficient number of deaths to allow robust estimates.

We included data on all 7·65 million deaths recorded in 2001–16 in our analyses, which were stratified by sex, 5-year age group, and decile of the Index of Multiple Deprivation (IMD). The IMD is the official measure of deprivation in England and is based on multiple indicators of individual and community welfare and wellbeing. Data were grouped into deciles of deprivation based on the ranked IMD scores of Lower Super Output Areas (LSOAs) in England in 2015; England is divided into 32 844 LSOAs, with a median LSOA population in 2015 of 1597 people. Decile 1 corresponds to the most deprived 10% of LSOAs (n=3284) and decile 10 to the most affluent 10% (n=3285). The most deprived LSOAs were mostly in urban areas, especially those in north and northeast England and the West Midlands—eg, Birmingham, Bradford, Leeds, Liverpool, Manchester, and Newcastle (appendix p 5). They were more frequently classified as urban conurbations than were the most affluent LSOAs (1936 [59%] of LSOAs in decile 1 *vs* 766 [23%] of LSOAs in decile 10), whereas the most affluent LSOAs were mostly in smaller cities and urban towns (1902 [58%]).

We used the same decile grouping throughout the study period for two reasons. First, consistent classification of LSOAs allows tracking of changes in death rates and life expectancy over time in consistent groups of LSOAs. Second, the IMD was calculated by the Office for National Statistics only for the years 2004, 2007, 2010, and 2015, which makes it impossible to use year-specific deciles. There was very high correlation (*r*= 0·95) between LSOA IMD ranks in 2004 and in 2015, with 89% of LSOAs (28 187 of the 31 726 LSOAs with consistent boundaries over time) being assigned to a decile in 2015 that was within one decile of that of 2004, and 99% (31 290 of 31 726) within two deciles. In other words, deprivation and affluence persist over time in England.

### Statistical methods

Division by sex, age group, decile of deprivation, and underlying cause of death can result in few deaths in some units, leading to highly variable estimates of death rates. To overcome this issue, we used a Bayesian hierarchical model to obtain estimates of death rates by sharing information across age groups and deprivation deciles and over time. In this approach, death rates for each age group, deprivation decile, and year are informed by data in that age group-deprivation-year unit as well as by those in the adjacent age groups, adjacent years, and adjacent deprivation deciles. The extent to which the estimated death rates are influenced by adjacent units depended on the number of deaths, with larger groups and those with higher death rates (eg, ischaemic heart disease in older ages) being largely informed by their own data and smaller groups with lower death rates (eg, some chronic diseases in young children) being influenced by the combination of their own data and data in other units.

The model was formulated to take into account how death rates vary in relation to age, time, and deprivation (appendix p 2). Specifically, we allowed each age group to have a different level (ie, intercept) and trend (ie, slope) in log-transformed death rate, and modelled age-group intercepts and slopes with a random-walk structure that is widely used to characterise smoothly varying age associations. This approach improves robustness of death rates in each age group and avoids implausible age patterns of mortality that could occur if each age group were analysed separately. We included similar terms and random-walk structures to describe the levels and trends of death rates in each deprivation decile. We also included age-deprivation interaction terms for both death rate level and trend. These terms allow the association of death rates with deprivation to vary by age group and, equivalently, each deprivation decile is able to have a different age pattern of mortality. Finally, because time trends in death rates can be non-linear, we modelled time trends of death rates using a linear term plus a smoothly varying non-linear term specified via a random walk. Detailed model specification is provided in the appendix and our statistical code can be downloaded online. All analyses were done separately by sex and for each cause of death because mortality levels and trends differ by sex and cause.

Age-standardised death rates were calculated using the age distribution of the combined female and male population of England in 2016. We calculated life expectancy at birth by decile of deprivation using life-table methods. We used the Kannisto-Thatcher method^6^ to expand the terminal age group (≥85 years) of the life table. We evaluated the performance of the model using the difference between life expectancies calculated from the observed death rates and those from the Bayesian hierarchical model. The median difference between observed and modelled life expectancy estimates across deprivation deciles and years was less than 0·001 years for females and for males and the median absolute differences were 0·045 years for females and 0·050 years for males; these differences remained similar across deprivation deciles and over time.

We calculated the contributions of deaths from each disease and injury, in each 5-year age group, to the life expectancy gap between the most deprived and affluent deciles using Arriaga's method, which is widely used to decompose life expectancy differences between populations or population subgroups.^7^ Arriaga's method calculates how much each age group contributes to the life expectancy gap by summing how much death rate differences at that age change the years of life lived both at that age and in subsequent ages through changing the number of survivors. It then uses information on how much each cause of death contributes to the differences in death rates between the deprived and affluent deciles to partition the age-specific contributions to the life expectancy gap by disease and injury.

All models were fitted using the R software (version 3.4.1) using integrated nested Laplace approximation, implemented in the R-INLA software. Further details are available in the appendix.

### Role of the funding source

The sponsor of the study had no role in study design, data collection and analysis, interpretation, or writing of the report. JEB had full access to all data used in this study. The corresponding author was responsible for submitting the Article for publication.

## Results

**Figure 1:**
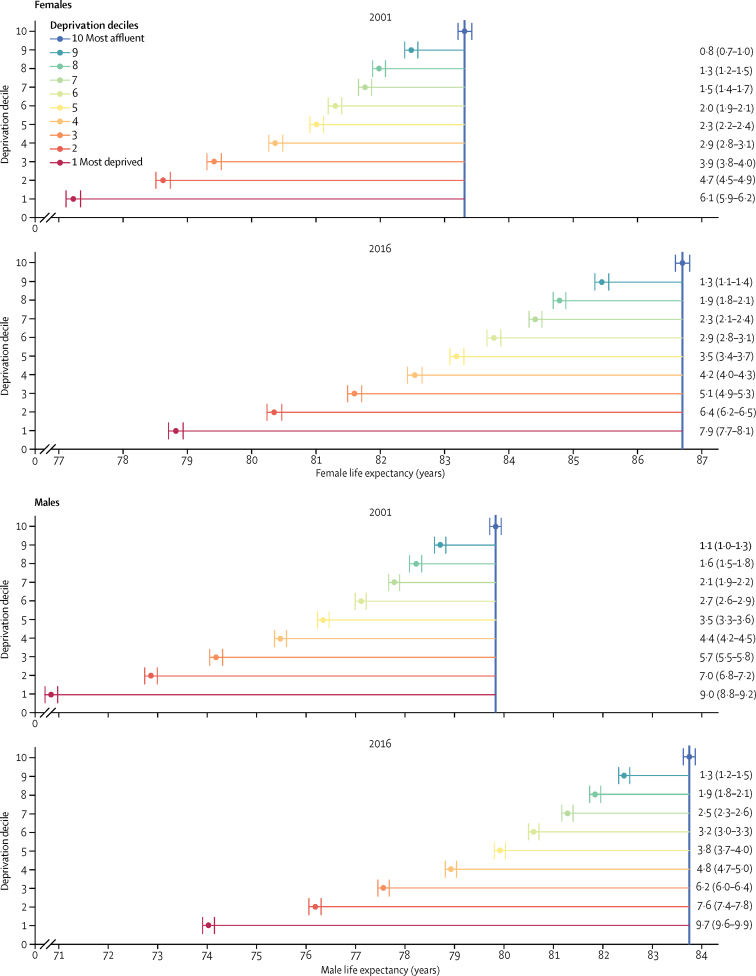
Life expectancy at birth by decile of deprivation and sex in 2001 and 2016 Point estimates of life expectancy for each estimate are shown, with credible intervals indicated by vertical bars. The numbers show the difference between life expectancy for each decile compared with that of the most affluent group, with credible intervals in brackets.

Life expectancy at birth in 2016 was consistently lower in more deprived communities, ranging from 78·8 years (95% credible interval 78·7–78·9) in the most deprived LSOAs to 86·7 years (86·6–86·8) in the most affluent ones for females and from 74·0 years (73·9–74·1) to 83·8 years (83·6–83·9) for males (figure 1). From decile 9 to decile 5, life expectancy dropped by about 6 months between adjacent deciles for both sexes (figure 1). However, the differences in life expectancy between deciles increased markedly both from decile 9 to decile 10, and for those below decile 5. At the extreme, the most deprived LSOAs were particularly badly off, with life expectancy lagging behind those of the next worst-off decile by 1·5 years (1·4–1·7) for females and 2·2 years (2·0–2·3) for males—a larger gap than between other adjacent deciles for both sexes.

Although life expectancy increased in every deprivation decile from 2001 to 2016, gains were larger in the better-off groups: 1·6 years (1·4–1·8) in the most deprived females compared with 3·4 years (3·2–3·5) in the least deprived females and 3·2 years (3·0–3·4) in the most deprived males compared with 3·9 years (3·7–4·1) in the least deprived males. As a result, the life expectancy gap between the most affluent and most deprived deciles increased from 6·1 years (5·9–6·2) in 2001 to 7·9 years (7·7–8·1) in 2016 in females and from 9·0 years (8·8–9·2) to 9·7 years (9·6–9·9) in males (figure 1). Since 2011, the rise in female life expectancy has reversed in the two most deprived deciles, declining by 0·24 years (0·10–0·37) in the most deprived and 0·16 years (0·02–0·29) in the second-most deprived by 2016, and has stalled in the third, fourth, and fifth most deprived deciles but has continued in better-off deciles with increases of up to 0·38 years in the more affluent deciles (appendix pp 7, 18).

**Figure 2:**
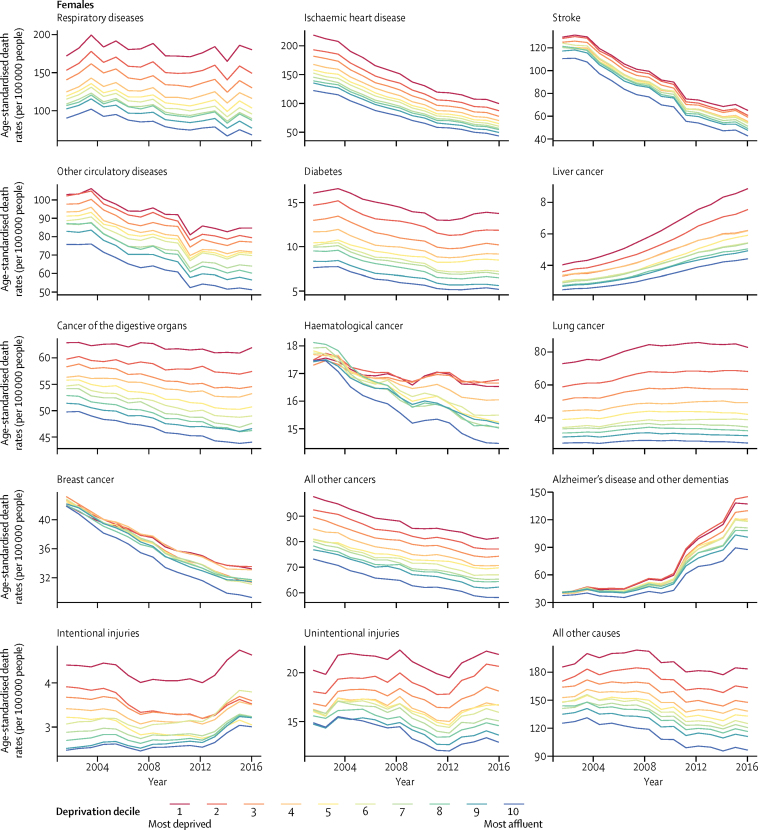

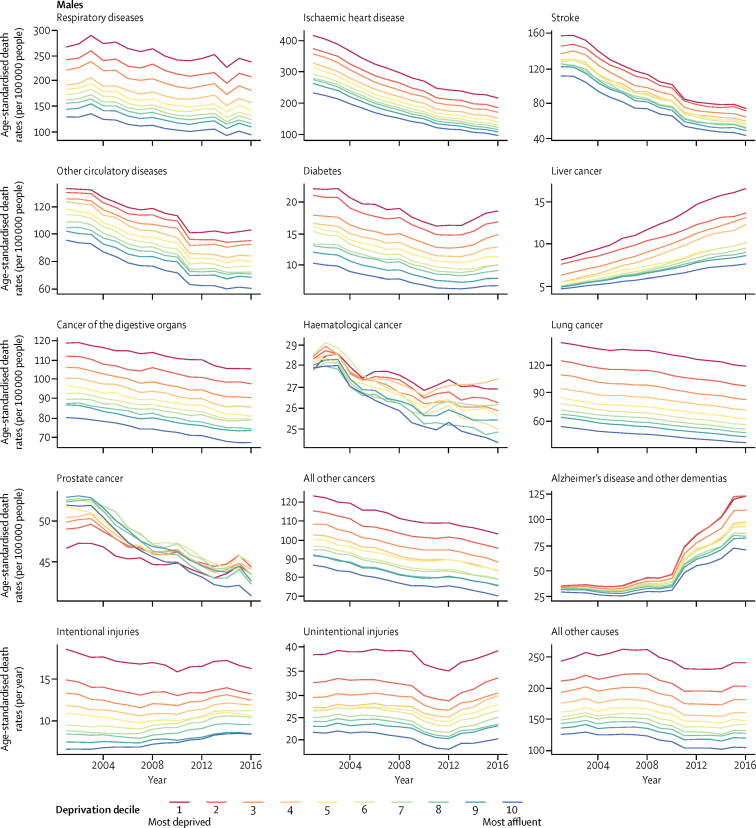
Trends in age-standardised death rates by underlying cause of death and decile of deprivation, by sex, from 2001 to 2016 Death rates are per 100 000 people per year. Numerical values for 2016 are given in the appendix (p 20).

Age-standardised death rates from all diseases and injuries were higher in the more deprived groups in most years, with death rates consistently increasing with deprivation for most diseases; the exceptions were haematological, breast, and prostate cancers in which we observed a slight reordering of death rates in comparison with deprivation (figure 2). Beyond the overall socioeconomic gradient, the most and sometimes second-most deprived groups stood separated from other groups in terms of having a higher age-standardised death rate for liver, digestive, and lung cancers; respiratory diseases; diabetes; intentional and unintentional injuries; and for a heterogeneous set of other causes of death. Absolute inequality (ie, difference) between the most and least deprived groups in 2016 was larger for diseases with higher age-standardised death rates—ie, ischaemic heart disease, respiratory diseases, lung cancer, and dementias. Relative inequality (ie, ratio) was largest for lung cancer, diabetes, and respiratory diseases, ranging from 2·5 to 3·3 in the two sexes; it was smallest for prostate, breast, and haematological cancers, with relative inequalities all at 1·1 (appendix p 20).

From 2001 to 2016, age-standardised death rates from most diseases declined, except for liver cancer and dementias for which death rates increased in every deprivation decile. Death rates from both intentional and unintentional injuries also increased after 2010 (with posterior probabilities greater than 0·80 of these recent increases being a true increase in all but two [intentional injuries] or three [unintentional injuries] of the 20 sex–deprivation decile combinations). Over the 16 years of analysis, absolute inequalities between the most and least deprived deciles increased for all diseases and injuries except ischaemic heart disease, intentional injuries, and the cluster of all other cancers in both sexes and stroke, lung cancer, and digestive cancers for men.

**Figure 3:**
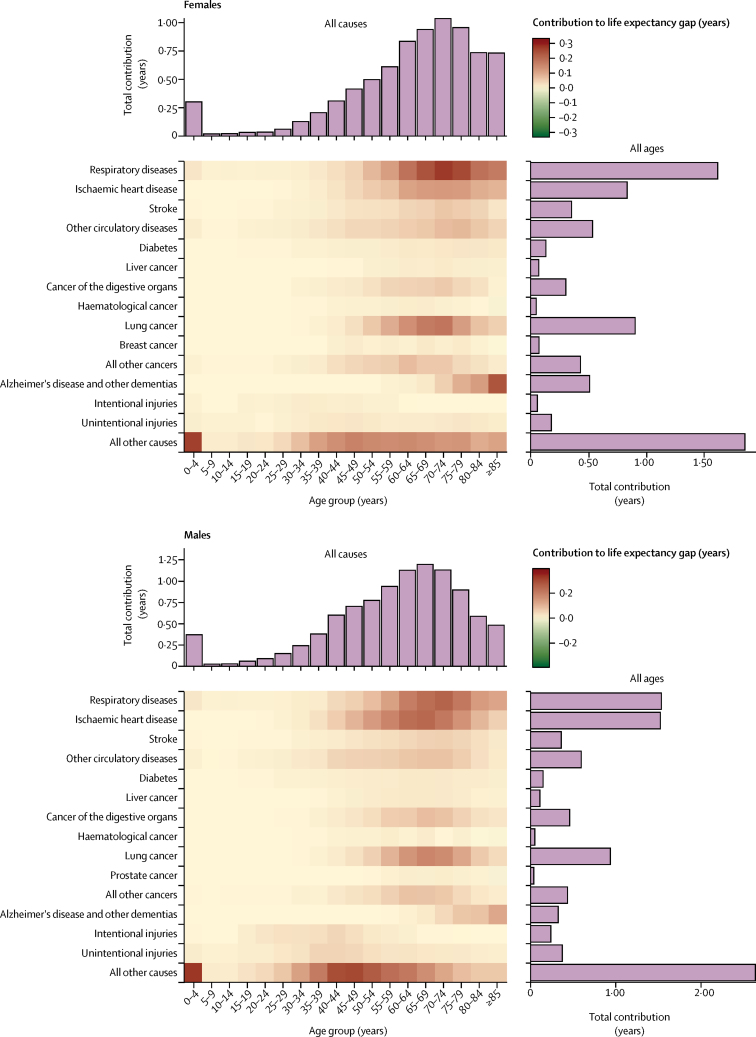
Contributions of deaths from different diseases and injuries at different ages to life expectancy inequality between the most affluent and most deprived deciles, by sex, 2016 See appendix (p 9) for summary of contributions from clusters of related causes.

For both females and males, 4% of the life expectancy gaps (0·3 of 7·9 years for females and 0·4 of 9·7 years for males) between the most affluent and most deprived groups in 2016 were due to differences in under-5 mortality, with about two-thirds of deaths in this age group occurring during the neonatal period. 6% (0·5 years) of the female gap and 10% (1·0 years) of the male gap were due to differences in those aged 5–39 years and 46% (3·6 years) of the female gap and 55% (5·3 years) of the male gap were due to differences in those aged 40–70 years; the remainder was due to differences among those aged 70 years and older (figure 3; appendix p 9). In addition to deaths of children younger than 5 years, major disease and injury contributors to social inequalities in life expectancy for both sexes were lung and digestive cancers (together contributing 1·2 years for females and 1·4 years for males), respiratory diseases (1·6 years and 1·5 years), ischaemic heart disease (0·8 years and 1·5 years), and dementias (0·5 years and 0·3 years, mostly above 70 years of age). Injuries also contributed to 0·2 years of the life expectancy gap between the most affluent and most deprived deciles in females and 0·6 years of the gap in males. No disease or injury had a negative contribution to inequalities at any age because death rates from every disease and injury were higher in the most deprived group than in the most affluent group at every age (figure 3).

**Figure 4:**
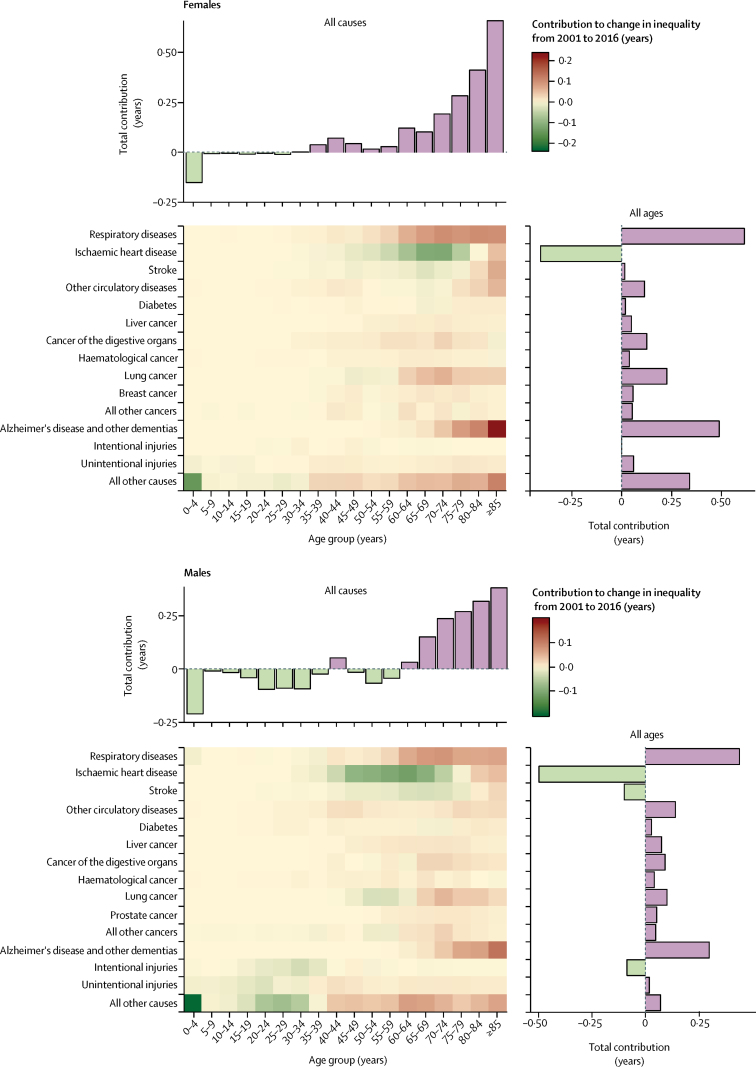
Change in the contributions of deaths from different diseases and injuries at different ages to life expectancy inequality between the most affluent and most deprived deciles, by sex, 2001–16 See appendix (p 12) for summary of contributions from clusters of related causes.

Although deaths in children younger than 5 years were a major contributor to life expectancy inequalities, their contribution declined from 2001 to 2016 from 0·5 years to 0·3 years for females and from 0·6 to 0·4 years for males (figure 4; appendix p 12) as absolute inequalities in under-5 mortality decreased. Both sexes experienced large declines in the contributions to life expectancy inequalities of ischaemic heart disease and, to a lesser extent, stroke, whereas men saw a large decline in the contribution of intentional injuries. The contributions of respiratory diseases, cancers, and dementias to the life expectancy gap between the affluent and deprived groups increased (figure 4; appendix p 12). The cancers whose contributions increased the most were liver and other digestive cancers for both sexes and lung and breast cancer for women. For men, the contributions of lung cancer to life expectancy inequalities fell below 65 years of age but increased in older ages, resulting in a net increase of about 0·1 years.

## Discussion

In this analysis of vital registration data from 2001 to 2016, we found that life expectancy increased for every decile of deprivation but the inequality between the deprived and affluent deciles grew larger. Importantly, since 2011, the rise in female life expectancy has reversed in the two most deprived deciles and stalled in the third, fourth, and fifth most deprived deciles, indicating that the poor are being entirely left out of the beneficial overall trends in life expectancy. The gap in female life expectancy between the most affluent and most deprived deciles of LSOAs is about the same as the difference between the UK's life expectancy as a whole (83·2 years) and that of Libya (75·0 years) or Azerbaijan (75·7 years), whereas the gap in male life expectancy is about the same as the difference between that of the UK as a whole (79·7 years) and that of Guatemala (70·4 years) or Azerbaijan (70·3 years).^8^ In 2016, the largest contributors to life expectancy inequalities between deprived and affluent areas of England were deaths in children younger than 5 years, dementias in older ages, and respiratory diseases, ischaemic heart disease, and a set of preventable and treatable cancers in working ages.

Our findings on rising inequalities in life expectancy in England are consistent with previous studies.^1^, ^2^ Similarly, our findings on inequalities in death rates and their trends are consistent with the few existing studies on specific diseases, such as cardiovascular diseases^9^, ^10^ and neonatal mortality.^11^ To our knowledge, only two consecutive annual reports^4^, ^5^ calculated the contribution of deaths from different age groups and from 11 broad clusters of diseases to life expectancy inequalities at one point in time, but they did not consider the changes in contributions over time nor the combination of disease and age groups. The large contributions of circulatory and respiratory diseases and cancers to the gap in life expectancy between the most and least deprived areas in those reports were consistent with our findings.

A strength of our study is that we report how clinically relevant causes of death at different ages contribute to current, and rising, life expectancy inequalities. We used a Bayesian model to robustly estimate death rates for different diseases by age group and deprivation decile. We grouped LSOAs by decile of IMD in 2015, which has the advantage of tracking the same groups of LSOAs over time but has the limitation of not reflecting historical inequalities if some LSOAs have switched deciles. Nonetheless, the rankings of LSOA IMD in 2015 were highly correlated with those in 2004 and few LSOAs moved by more than one decile between these years. Furthermore, the population of each LSOA might change because of migration, both within the country and overseas.^12^ Therefore, life expectancy trends should not be attributed solely to changes in health status of individuals. Studies in the UK^12^, ^13^ have shown that migration alone does not explain trends in health and health inequalities and that these trends represent real changes in population health. Even if rising inequalities are partly due to migration (often by healthy people) from one area to another, such migration patterns have social and economic roots that should be addressed through employment opportunities, affordable housing, and high-quality education and health care. Finally, even though LSOAs have small populations, there are inevitably variations in socioeconomic status and health within them. From the perspective of interventions, this heterogeneity means that there is a need for interventions based on individual attributes (eg, social benefits or targeted financial incentives for healthy foods) as well as those that address communities (eg, more equitable provision of primary and specialist care), as we discuss below.

Policies in the 1970s and 1980s that reduced job security, increased unemployment, and worsened income inequalities in the UK were important determinants of rising health inequalities in subsequent decades.^14^ Today, once again, despite historic low levels of formal unemployment, working incomes have stagnated for the poorest^15^ and the new gig economy has diminished job security.^16^ Low-wage employment, coupled with unprecedented cuts to in-work and out-of-work benefits,^17^ has contributed to 1·5 million people experiencing destitution,^18^ with particularly large effects in children and people of working age in already disadvantaged groups.^19^, ^20^, ^21^, ^22^, ^23^, ^24^, ^25^ As described in the *The New York Times*, “after eight years of budget cutting, Britain is looking less like the rest of Europe and more like the United States, with a shrinking welfare state and spreading poverty”.^26^

Smoking, alcohol use, and poor nutrition, which all have substantial social inequalities, are important causes of some of the diseases with the largest contributions to life expectancy inequalities. Given the social inequalities associated with these risk factors, some public health efforts addressing them might have inadvertently worsened inequalities even if in some cases, such as smoking, they have helped to reduce mortality for all social groups, including the most deprived. In particular, social inequalities in smoking have increased as aggregate prevalence has declined,^4^ especially for smoking during pregnancy, which has a more than ten-fold variation in prevalence among English local authorities.^27^ Substantial reductions in local authority smoking cessation budgets risk worsening these inequalities.

Fiscal and regulatory measures tend to be more effective at achieving behavioural change and reducing inequalities than voluntary approaches.^28^ For example, the UK's regulatory approach to salt reformulation under the Food Standards Agency's leadership achieved a 15% reduction in salt consumption up to 2010 through enforcement of targets to reduce salt content in processed foods. By contrast, the voluntary and self-policing approach of the failed Public Health Responsibility Deal (which has since been abandoned) stalled this progress from 2010 onwards.^29^ Given these experiences, the UK should embrace fiscal and regulatory policies for alcohol and foods rich in sugar, salt, and trans fats. Finally, and importantly, the cost of healthy foods, especially fresh fruits and vegetables, has increased in the UK and other high-income countries^30^ relative to unhealthy processed foods. Coupled with the rising number of families on benefits and working families needing to use foodbanks because their income does not cover the cost of essentials,^31^ substantial improvements in diet require making healthier foods affordable to poor families through targeted financial mechanisms.^32^

Finally, our finding that death rates from every disease and at every age were higher in deprived areas than in affluent ones means that access to and utilisation of high-quality health care is an essential component of strategies for reducing life expectancy inequalities. For example, more equitable treatment of acute coronary events and secondary prevention in survivors^33^ are likely to have contributed to declining absolute inequalities in ischaemic heart disease mortality.

The potential of universal high-quality health care to reduce health inequalities is undermined by unequal provision of health care and unequal opportunities for its utilisation, leading to worse survival in deprived groups compared with affluent ones. For example, patients from deprived areas have a later cancer diagnosis and worse survival than their more affluent counterparts.^34^

Three components of health and social care are particularly relevant for reducing inequalities under the principle of proportionate universalism: access to health care; health promotion and disease prevention through risk prediction and early diagnosis; and strengthening integrated health and social care. First, although universal access is a pillar of the UK National Health Service (NHS), in practice services are not equally accessible due to how they are provided and used. In terms of provision, previous efforts to reduce inequalities have had some success in increasing physician numbers in specific deprived areas.^35^ However, health and social care is now more stretched than ever before, owing to a lengthy period of below-average annual funding increases since 2010 (1·0% for health compared with a long-term average of 3·9%). The funding squeeze, a growing and ageing population, and workforce shortages have worsened wait times for primary and specialist care, especially in deprived areas.^33^, ^36^ Unequal utilisation of services is multifaceted but limited accessibility outside of working hours coupled with little or no flexibility in terms of work hours or schedule^37^ and transport cost and time are important obstacles to use in deprived groups.

Screening programmes such as the NHS Health Check are effective at identifying those at high risk of adverse health events for some of the diseases with large contributions to life expectancy inequalities. Screening, although beneficial for all social groups, risks worsening inequalities if it differentially benefits more affluent groups. Making screening more equitable is partly related to health-care access and utilisation, as discussed above. Novel point-of-care technologies, such as those for cancer detection,^38^ have the potential to move screening from clinic and laboratory into homes and communities. However, equity must be integral to the rollout of new diagnostic technologies to avoid exacerbating existing inequalities through a two-tier system of access and utilisation.

Finally, curbing and reversing the worsening inequalities in chronic conditions such as dementia requires enhancement and better integration of health and social care. This objective is not only hindered but also set back by the substantial cuts to local authority budgets over the past 8 years, resulting in worse outcomes for the more deprived groups compared with those able to pay for better care.^39^, ^40^

To reduce health inequalities, sustained and coordinated action is needed across the three areas of economic and social determinants, risk behaviours and environments, and health and social care.^3^, ^35^ The UK now faces a perfect storm of obstacles to reducing health inequalities, with all three areas simultaneously in a condition of policy stagnation and regression. A positive tide might be emerging, however, as, after decades of being treated as secondary to aggregate improvements in the economy and health, inequalities are emerging as the central issue in policy and political debates in the UK and other high-income countries. Pressured by the public, civil society organisations, and philanthropists, political parties—while differing massively in their ideology and responses—are acknowledging inequalities as a key social challenge and promising policy responses. Health equity in all policies should become a key aim of these responses.

For the **statistical code** see http://globalenvhealth.org/code-data-download

## Data sharing
