# Supplementary material for: Contributions of diseases and injuries to widening life expectancy inequalities in England from 2001 to 2016: a population-based analysis of vital registration data
Source: Lancet Public Health. 2018 Nov 23;3(12):e586–97. doi: 10.1016/S2468-2667(18)30214-7 (PMC6277818; doi:10.1016/S2468-2667(18)30214-7)
Supplement: Supplementary appendix [file mmc1.pdf]

THE LANCET
Public Health

Supplementary appendix

This appendix formed part of the original submission and has been peer reviewed. We post it as supplied by the authors.

Supplement to: Bennett JE, Pearson-Stuttard J, Kontis V, Capewell S, Wolfe I, Ezzati M. Contributions of diseases and injuries to widening life expectancy inequalities in England from 2001 to 2016: a population-based analysis of vital registration data. *Lancet Public Health* 2018; published online Nov 22. http://dx.doi.org/10.1016/S2468-2667(18)30214-7.

Webappendix

Table of contents

Appendix Text: Statistical methods 2

References.. 4

Appendix Figure 1: Map of Lower Super Output Areas (LSOA) by decile of deprivation in 2015. 5

Appendix Figure 2: Trends in life expectancy at birth by sex and decile of deprivation from 2001 to 2016.. 7

Appendix Figure 3: Contributions of deaths from seven broad disease and injury groups at different ages to life expectancy inequality between the most affluent and most deprived deciles, 2016. 9

Appendix Figure 4: Change in the contributions of deaths from seven broad disease and injury groups at different ages to life expectancy inequality between the most affluent and most deprived deciles, 2001–16.. 12

Appendix Table 1: Clusters of causes of death used in the analysis with ICD codes..... 15

Appendix Table 2: Life expectancy at birth by decile of Index of Multiple Deprivation (IMD), year and sex. 18

Appendix Table 3: Age standardised death rates (per 100,000 people) in 2016 by cause of death, decile of Index of Multiple Deprivation (IMD), and sex.................................. 20

Appendix Text: Statistical methods

As described in the main text, we used a Bayesian hierarchical model to obtain robust estimates of death rates by age group, deprivation decile and year, which were then used to calculate life expectancy and age standardised death rates. This model was formulated to incorporate established features of death rates in relation to age and deprivation, and over time. Specifically, for each sex and cause of death, the number of deaths in age group a $(= 1, \ldots, 18)$, deprivation decile d $(= 1, \ldots, 10)$ and year t $(= 2001, \ldots, 2016)$ follows a Poisson distribution

$$\text{Deaths}_{adt} \sim \text{Poisson}(m_{adt} \cdot \text{Population}_{adt}) \, ,$$

where m_{adt} is the death rate. Log-transformed death rates were allowed to have different levels and trends that depend on time, age group and deprivation decile while borrowing strength across age groups and deprivation, as described below.

The model contains terms to capture the overall level and rate of change of mortality, as well as age-specific and deprivation decile-specific terms that allow each age group's and deprivation decile's mortality level and trend to deviate from the common terms. Log-transformed death rates are modelled as

$$\log(m_{at}) = \alpha_0 + \alpha_a + \alpha_d + \alpha_{ad} + \beta_0 . t + \beta_a . t + \beta_{\mathrm{d}} . t + \beta_{ad} . t + \nu_{at} + \nu_{dt} + \varepsilon_{adt} \, ,$$

where α_0 is the common intercept for death rates across age groups and deprivation deciles; β_0 quantifies the common trend across ages and deprivation deciles. α_a and β_a measure deviation from the common level and trend, respectively, by age group. α_d and β_d measure deviation from the common level and trend, respectively, by deprivation decile. α_{ad} and β_{ad} are age group-deprivation decile interaction terms which measure age-specific deviations in the level and trend of mortality in deprivation decile d from that of other deciles. These interaction terms allow the role of deprivation to depend on age group, and vice versa. Interaction terms were modelled as $\mathcal{N}(0, \sigma^2_{\alpha_{ad}})$ and $\mathcal{N}(0, \sigma^2_{\beta_{ad}})$ respectively.

Borrowing strength and smoothness over adjacent age groups and adjacent deprivation deciles was done using first-order random walk priors on α_a, α_d, β_a and β_{d}. The first-order random walk prior takes a general form of, for example, $\alpha_a \sim \mathcal{N}(\alpha_{a-1}, \sigma^2_{\alpha_a})$. For each age a, the term ν_{at} is a first-order random walk over time that allows for nonlinearity in the age trends, beyond what is accounted for by other trend components. The degree of correlation among these age-specific random walks is modelled via a parameter ρ_a that is estimated by the model, as detailed in a methodological reference.[1] ν_{dt} and ρ_d are corresponding terms which allow for nonlinearity in the deprivation decile trends.

The ε_{adt}, modelled as $\mathcal{N}(0, \sigma^2_{\varepsilon})$, account for additional variability in the data not captured by other components in the models.

Hyper-priors

We used weakly informative priors so that parameter estimation was driven by the data.[2] The hyper-priors were defined on the logarithm of the precisions of the random effects, for example on $\log(1/\sigma_\alpha^2)$. These were modelled as $\text{logGamma}(\theta, \gamma)$ distributions with shape $\theta = 1$ and rate $\gamma = 0.001$. The same hyper-priors were used for all precision parameters of the random effects in the model described above. For the common slopes and intercepts, we used $\mathcal{N}(0, 1000)$.

Implementation

All models were fitted using the R software (version 3.4.1).[3] All models were fitted using integrated nested Laplace approximation,[4] implemented in the R-INLA software (version 0.0-1455098891).[5]

References

1. Riebler A, Held L, Rue H. Estimation and extrapolation of time trends in registry data - Borrowing strength from related populations. *Ann Appl Stat* 2012; **6**(1): 304-33.
2. Gelman A, Carlin JB, Stern HS, Dunson DB, Vehtari A, Rubin DB. Bayesian Data Analysis, Third Edition. Boca Raton: Chapman and Hall/CRC 2013.
3. R Core Team. R: A language and environment for statistical computing. Vienna, Austria: R Foundation for Statistical Computing; 2015.
4. Rue H, Martino S, Chopin N. Approximate Bayesian inference for latent Gaussian models by using integrated nested Laplace approximations. *J Roy Stat Soc B* 2009; **71**: 319-92.
5. Lindgren F, Rue H. Bayesian spatial modelling with R-INLA. *J Stat Softw* 2015; **63**(19): 1-25.

Appendix Figure 1: Map of Lower Super Output Areas (LSOA) by decile of deprivation in 2015.

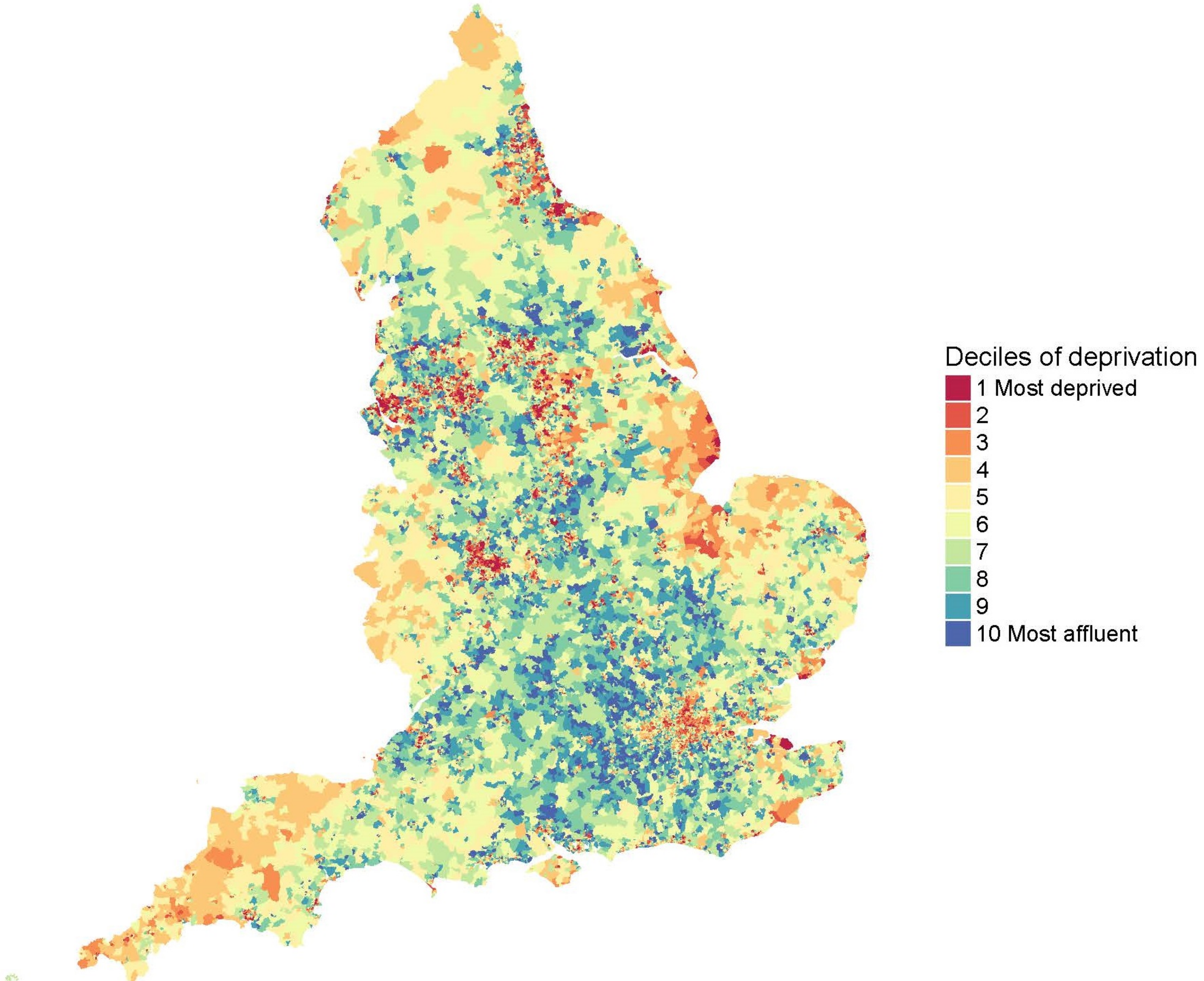
Deciles of deprivation
1 Most deprived
2
3
4
5
6
7
8
9
10 Most affluent

Appendix Figure 2: Trends in life expectancy at birth by sex and decile of deprivation from 2001 to 2016.

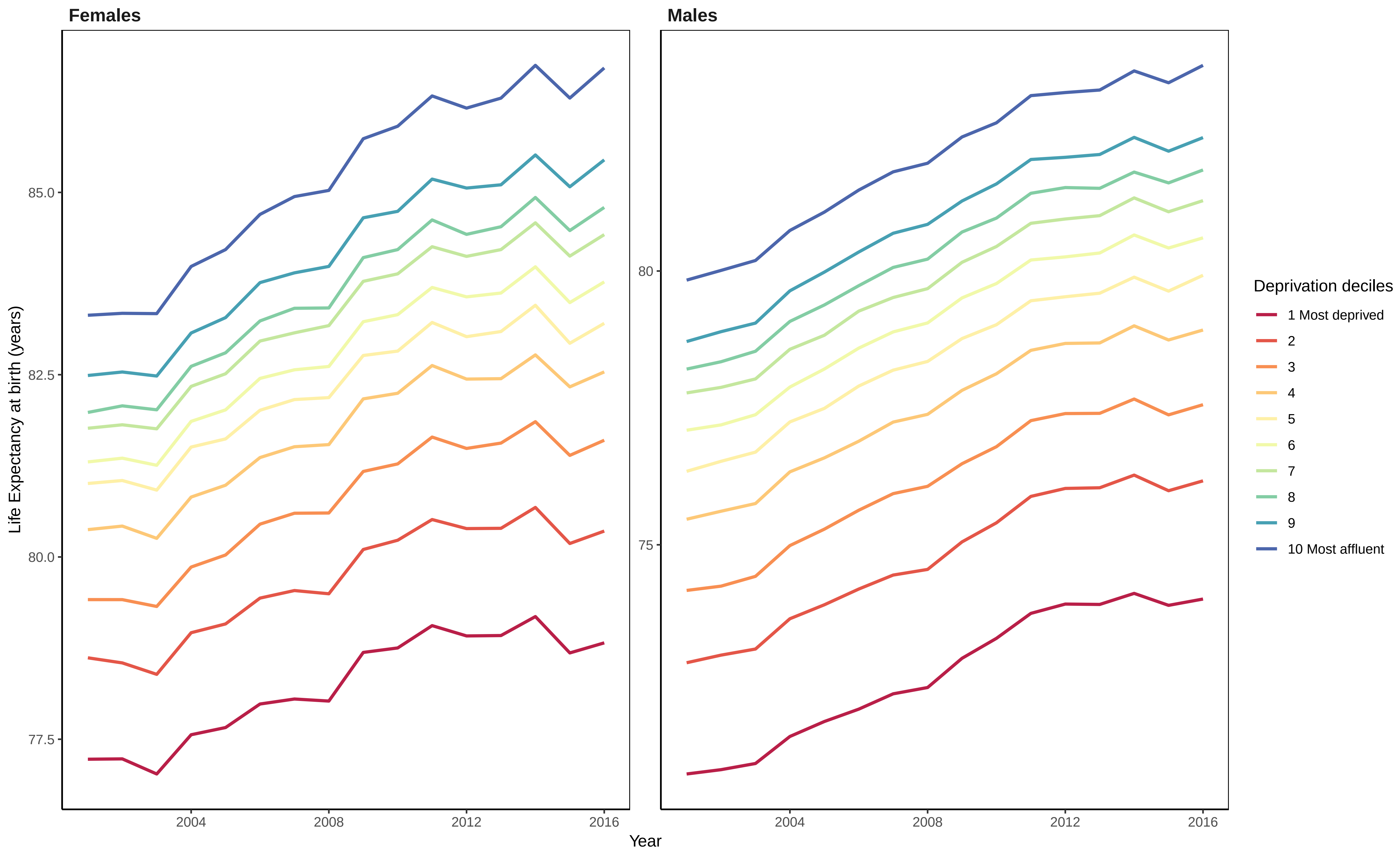
Females
Males
85.0
82.5
80.0
77.5
80
75
2004
2008
2012
2016
Life Expectancy at birth (years)
Year
Deprivation deciles
1 Most deprived
2
3
4
5
6
7
8
9
10 Most affluent

Appendix Figure 3: Contributions of deaths from seven broad disease and injury groups at different ages to life expectancy inequality between the most affluent and most deprived deciles in 2016.

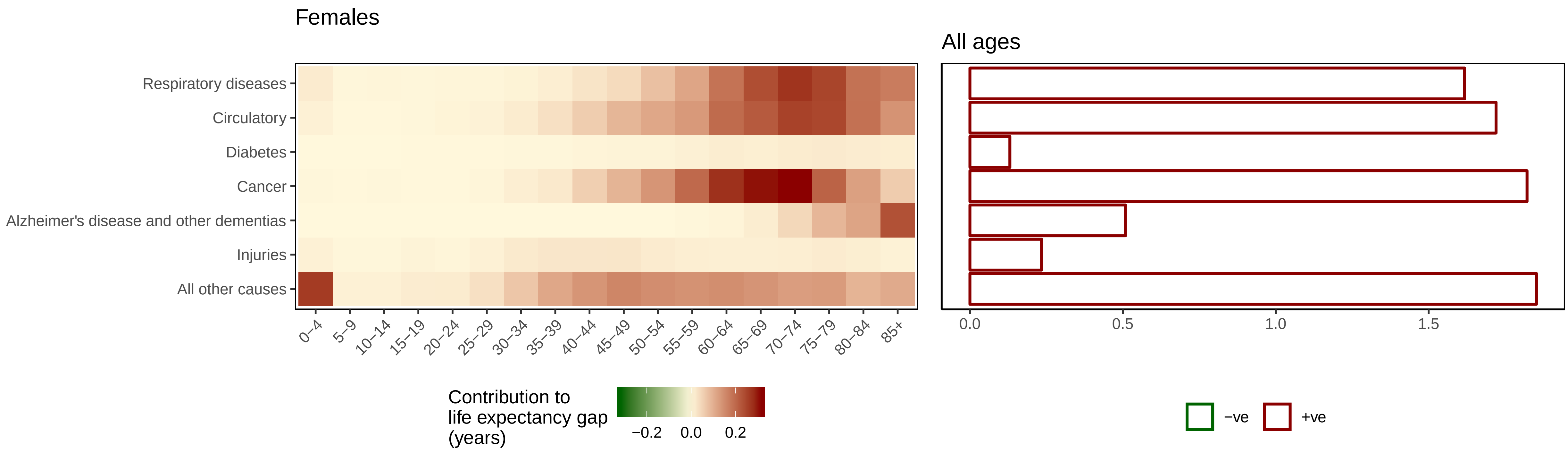

Females
All ages
Respiratory diseases
Circulatory
Diabetes
Cancer
Alzheimer's disease and other dementias
Injuries
All other causes
0–4
5–9
10–14
15–19
20–24
25–29
30–34
35–39
40–44
45–49
50–54
55–59
60–64
65–69
70–74
75–79
80–84
85+
0.0
0.5
1.0
1.5
Contribution to life expectancy gap (years)
−0.2
0.0
0.2
−ve
+ve

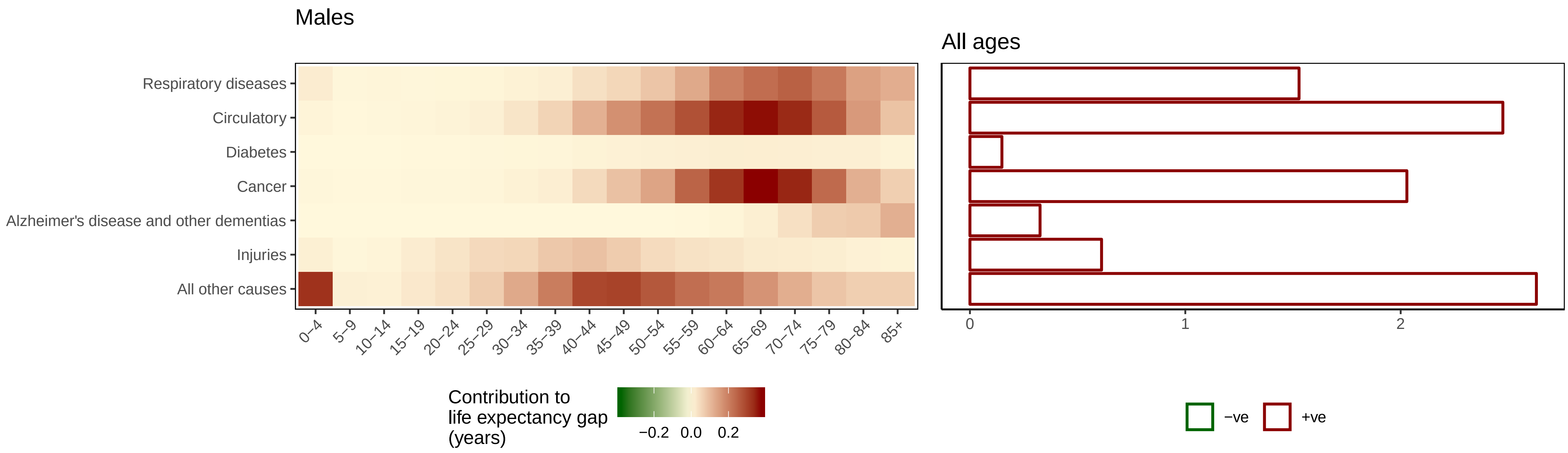
Males
All ages
Respiratory diseases
Circulatory
Diabetes
Cancer
Alzheimer's disease and other dementias
Injuries
All other causes
0–4
5–9
10–14
15–19
20–24
25–29
30–34
35–39
40–44
45–49
50–54
55–59
60–64
65–69
70–74
75–79
80–84
85+
0
1
2
Contribution to life expectancy gap (years)
−0.2
0.0
0.2
−ve
+ve

Appendix Figure 4: Change in the contributions of deaths from seven broad disease and injury groups at different ages to life expectancy inequality between the most affluent and most deprived deciles, 2001–16.

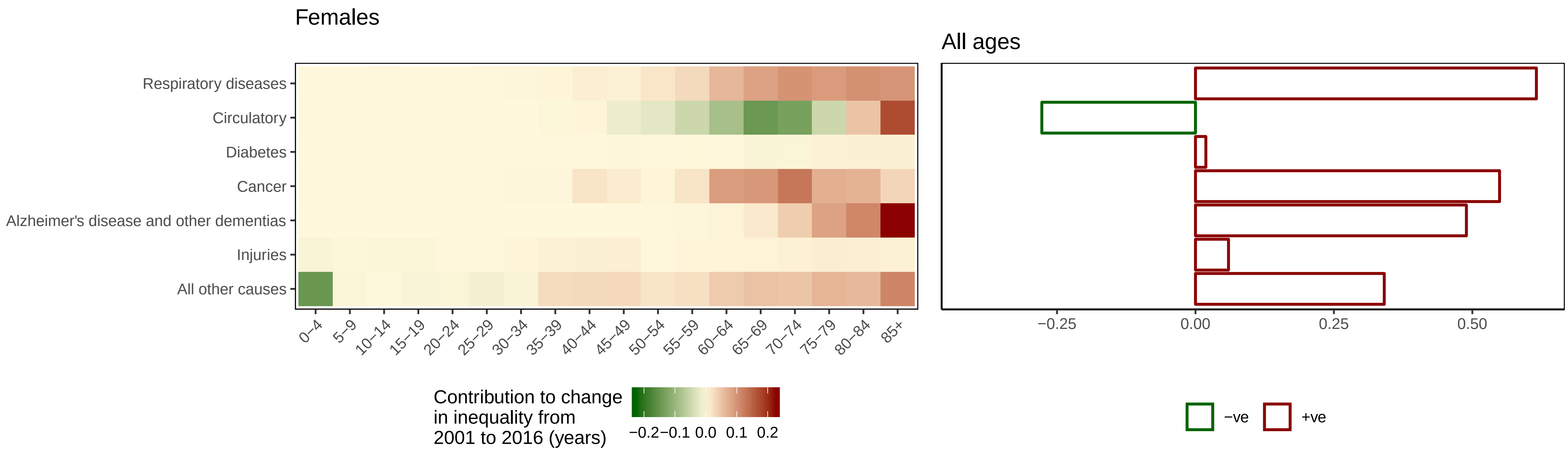
Females
All ages
Respiratory diseases
Circulatory
Diabetes
Cancer
Alzheimer's disease and other dementias
Injuries
All other causes
0–4
5–9
10–14
15–19
20–24
25–29
30–34
35–39
40–44
45–49
50–54
55–59
60–64
65–69
70–74
75–79
80–84
85+
−0.25
0.00
0.25
0.50
Contribution to change in inequality from 2001 to 2016 (years)
−0.2
−0.1
0.0
0.1
0.2
−ve
+ve

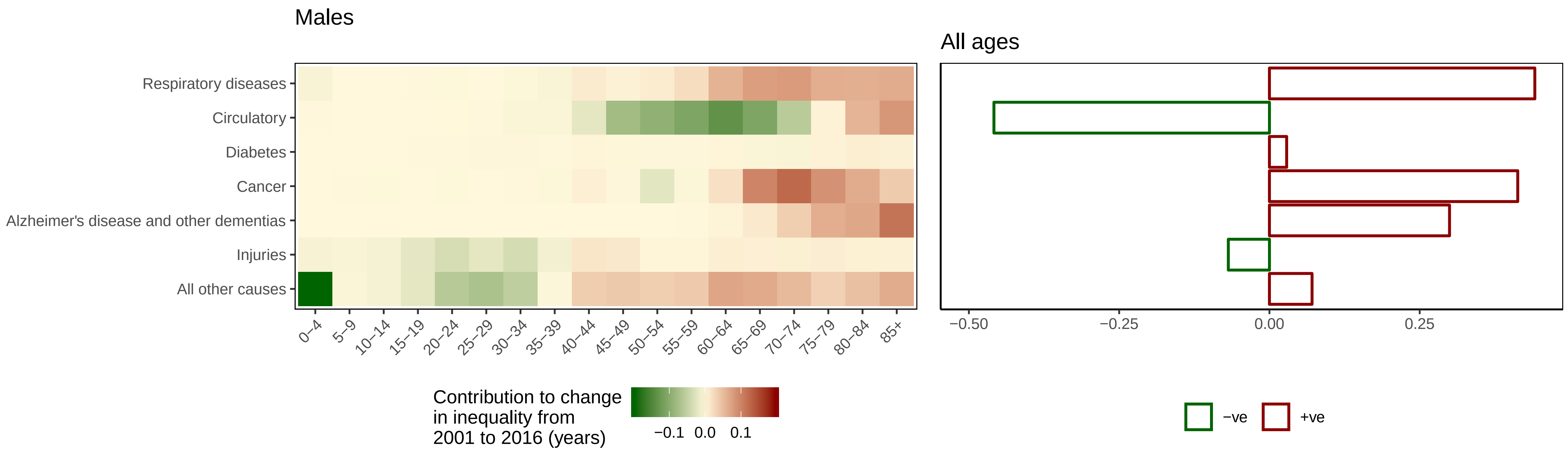
Males
All ages
Respiratory diseases
Circulatory
Diabetes
Cancer
Alzheimer's disease and other dementias
Injuries
All other causes
0–4
5–9
10–14
15–19
20–24
25–29
30–34
35–39
40–44
45–49
50–54
55–59
60–64
65–69
70–74
75–79
80–84
85+
−0.50
−0.25
0.00
0.25
Contribution to change in inequality from 2001 to 2016 (years)
−0.1
0.0
0.1
−ve
+ve

Appendix Table 1: Clusters of causes of death used in the analysis with ICD codes.

Disease group	ICD-10 codes
**Diseases of the respiratory system**	J00-J99
**Ischemic heart disease**	I20-I25
**Stroke/cerebrovascular disease**	I60-I69
**Other circulatory diseases**	I00-I19, I26-I59, I70-I99
**Diabetes**	E10-E14
**Cancer of the liver and intrahepatic bile ducts**	C22
**Cancer of digestive organs (except liver and intrahepatic bile ducts)**	C15-C21, C23-C26
**Cancer of lymphoid, haematopoietic and related tissue**	C81-C96
**Cancer of trachea, bronchus and lung**	C33-C34
**Cancer of prostate**	C61
**Cancer of breast***	C50
**All other cancers**	C00-C14, C27-C32, C35-C49, C51-C60, C62-C80, C97-D48
**Alzheimer's disease and other dementias**	F00-F03, G30
**Intentional injuries**	X60, X61, X62, X63, X64, X65, X66, X67, X68, X69, X70, X71, X72, X73, X74, X741, X742, X743, X744, X749, X75, X76, X77, X78, X79, X80, X81, X82, X83, X84, X85, X86, X87, X88, X89, X90, X91, X92, X93, X94, X95, X951, X952, X953, X954, X959, X96, X97, X98, X99, Y00, Y01, Y02, Y03, Y04, Y05, Y06, Y06.0, Y06.1, Y06.2, Y06.8, Y06.9, Y07, Y07.0, Y07.1, Y07.2, Y07.3, Y07.8, Y07.9, Y08, Y09, Y35, Y35.0, Y35.1, Y35.2, Y35.3, Y35.4, Y35.5, Y35.6, Y35.7, U01.1, Y36, Y36.0, Y36.1, Y36.2, Y36.3, Y36.4, Y36.5, Y36.6, Y36.7, Y36.8, Y36.9, Y87.0, Y87.1, Y89.0, Y89.1
**Unintentional injuries**	V01, V01.0, V01.1, V01.9, V02, V02.0, V02.1, V02.9, V03, V03.0, V03.1, V03.9, V04, V04.0, V04.1, V04.9, V05, V05.0, V05.1, V05.9, V06, V06.0, V06.1, V06.9, V09, V09.0, V09.1, V09.2, V09.3, V09.9, V10, V10.0, V10.1, V10.2, V10.3, V10.4, V10.5, V10.9, V11, V11.0, V11.1, V11.2, V11.3, V11.4, V11.5, V11.9, V12, V12.0, V12.1, V12.2, V12.3, V12.4, V12.5, V12.9, V13, V13.0, V13.1, V13.2, V13.3, V13.4, V13.5, V13.9, V14, V14.0, V14.1, V14.2, V14.3, V14.4, V14.5, V14.9, V15, V15.0, V15.1, V15.2, V15.3, V15.4, V15.5, V15.9, V16, V16.0, V16.1, V16.2, V16.3, V16.4, V16.5, V16.9, V17, V17.0, V17.1, V17.2, V17.3, V17.4, V17.5, V17.9, V18, V18.0, V18.1, V18.2, V18.3, V18.4, V18.5, V18.9, V19, V19.0, V19.1, V19.2, V19.3, V19.4, V19.5, V19.6, V19.8, V19.9, V20, V20.0, V20.1, V20.2, V20.3, V20.4, V20.5, V20.9, V21, V21.0, V21.1, V21.2, V21.3, V21.4, V21.5, V21.9, V22, V22.0, V22.1, V22.2, V22.3, V22.4, V22.5, V22.9, V23, V23.0, V23.1, V23.2, V23.3, V23.4, V23.5, V23.9, V24, V24.0, V24.1, V24.2, V24.3, V24.4, V24.5, V24.9, V25, V25.0, V25.1, V25.2, V25.3, V25.4, V25.5, V25.9, V26, V26.0, V26.1, V26.2, V26.3, V26.4, V26.5, V26.9, V27, V27.0, V27.1, V27.2, V27.3, V27.4, V27.5, V27.9, V28, V28.0, V28.1, V28.2, V28.3, V28.4, V28.5, V28.9, V29, V29.0, V29.1, V29.2, V29.3, V29.4, V29.5, V29.6, V29.8, V29.9, V30, V30.0, V30.1, V30.2, V30.3, V30.4, V30.5, V30.6, V30.7, V30.9, V31, V31.0, V31.1, V31.2, V31.3, V31.4, V31.5, V31.6, V31.7, V31.9, V32, V32.0, V32.1, V32.2, V32.3, V32.4, V32.5, V32.6, V32.7, V32.9, V33, V33.0, V33.1, V33.2, V33.3, V33.4, V33.5, V33.6, V33.7, V33.9, V34, V34.0, V34.1, V34.2, V34.3, V34.4,

	V34.5, V34.6, V34.7, V34.9, V35, V35.0, V35.1, V35.2, V35.3, V35.4, V35.5, V35.6, V35.7, V35.9, V36, V36.0, V36.1, V36.2, V36.3, V36.4, V36.5, V36.6, V36.7, V36.9, V37, V37.0, V37.1, V37.2, V37.3, V37.4, V37.5, V37.6, V37.7, V37.9, V38, V38.0, V38.1, V38.2, V38.3, V38.4, V38.5, V38.6, V38.7, V38.9, V39, V39.0, V39.1, V39.2, V39.3, V39.4, V39.5, V39.6, V39.8, V39.9, V40, V40.0, V40.1, V40.2, V40.3, V40.4, V40.5, V40.6, V40.7, V40.9, V41, V41.0, V41.1, V41.2, V41.3, V41.4, V41.5, V41.6, V41.7, V41.9, V42, V42.0, V42.1, V42.2, V42.3, V42.4, V42.5, V42.6, V42.7, V42.9, V43, V43.0, V43.1, V43.2, V43.3, V43.4, V43.5, V43.6, V43.7, V43.9, V44, V44.0, V44.1, V44.2, V44.3, V44.4, V44.5, V44.6, V44.7, V44.9, V45, V45.0, V45.1, V45.2, V45.3, V45.4, V45.5, V45.6, V45.7, V45.9, V46, V46.0, V46.1, V46.2, V46.3, V46.4, V46.5, V46.6, V46.7, V46.9, V47, V47.0, V47.1, V47.2, V47.3, V47.4, V47.5, V47.6, V47.7, V47.9, V48, V48.0, V48.1, V48.2, V48.3, V48.4, V48.5, V48.6, V48.7, V48.9, V49, V49.0, V49.1, V49.2, V49.3, V49.4, V49.5, V49.6, V49.8, V49.9, V50, V50.0, V50.1, V50.2, V50.3, V50.4, V50.5, V50.6, V50.7, V50.9, V51, V51.0, V51.1, V51.2, V51.3, V51.4, V51.5, V51.6, V51.7, V51.9, V52, V52.0, V52.1, V52.2, V52.3, V52.4, V52.5, V52.6, V52.7, V52.9, V53, V53.0, V53.1, V53.2, V53.3, V53.4, V53.5, V53.6, V53.7, V53.9, V54, V54.0, V54.1, V54.2, V54.3, V54.4, V54.5, V54.6, V54.7, V54.9, V55, V55.0, V55.1, V55.2, V55.3, V55.4, V55.5, V55.6, V55.7, V55.9, V56, V56.0, V56.1, V56.2, V56.3, V56.4, V56.5, V56.6, V56.7, V56.9, V57, V57.0, V57.1, V57.2, V57.3, V57.4, V57.5, V57.6, V57.7, V57.9, V58, V58.0, V58.1, V58.2, V58.3, V58.4, V58.5, V58.6, V58.7, V58.9, V59, V59.0, V59.1, V59.2, V59.3, V59.4, V59.5, V59.6, V59.8, V59.9, V60, V60.0, V60.1, V60.2, V60.3, V60.4, V60.5, V60.6, V60.7, V60.9, V61, V61.0, V61.1, V61.2, V61.3, V61.4, V61.5, V61.6, V61.7, V61.9, V62, V62.0, V62.1, V62.2, V62.3, V62.4, V62.5, V62.6, V62.7, V62.9, V63, V63.0, V63.1, V63.2, V63.3, V63.4, V63.5, V63.6, V63.7, V63.9, V64, V64.0, V64.1, V64.2, V64.3, V64.4, V64.5, V64.6, V64.7, V64.9, V65, V65.0, V65.1, V65.2, V65.3, V65.4, V65.5, V65.6, V65.7, V65.9, V66, V66.0, V66.1, V66.2, V66.3, V66.4, V66.5, V66.6, V66.7, V66.9, V67, V67.0, V67.1, V67.2, V67.3, V67.4, V67.5, V67.6, V67.7, V67.9, V68, V68.0, V68.1, V68.2, V68.3, V68.4, V68.5, V68.6, V68.7, V68.9, V69, V69.0, V69.1, V69.2, V69.3, V69.4, V69.5, V69.6, V69.8, V69.9, V70, V70.0, V70.1, V70.2, V70.3, V70.4, V70.5, V70.6, V70.7, V70.9, V71, V71.0, V71.1, V71.2, V71.3, V71.4, V71.5, V71.6, V71.7, V71.9, V72, V72.0, V72.1, V72.2, V72.3, V72.4, V72.5, V72.6, V72.7, V72.9, V73, V73.0, V73.1, V73.2, V73.3, V73.4, V73.5, V73.6, V73.7, V73.9, V74, V74.0, V74.1, V74.2, V74.3, V74.4, V74.5, V74.6, V74.7, V74.9, V75, V75.0, V75.1, V75.2, V75.3, V75.4, V75.5, V75.6, V75.7, V75.9, V76, V76.0, V76.1, V76.2, V76.3, V76.4, V76.5, V76.6, V76.7, V76.9, V77, V77.0, V77.1, V77.2, V77.3, V77.4, V77.5, V77.6, V77.7, V77.9, V78, V78.0, V78.1, V78.2, V78.3, V78.4, V78.5, V78.6, V78.7, V78.9, V79, V79.0, V79.1, V79.2, V79.3, V79.4, V79.5, V79.6, V79.8, V79.9, V80, V80.0, V80.1, V80.2, V80.3, V80.4, V80.5, V80.6, V80.7, V80.8, V80.9, V81, V81.0, V81.1, V81.2, V81.3, V81.4, V81.5, V81.6, V81.7, V81.8, V81.9, V82, V82.0, V82.1, V82.2, V82.3, V82.4, V82.5, V82.6, V82.7, V82.8, V82.9, V83, V83.0, V83.1, V83.2, V83.3, V83.4, V83.5, V83.6, V83.7, V83.9, V84, V84.0, V84.1, V84.2, V84.3, V84.4, V84.5, V84.6, V84.7, V84.9, V85, V85.0, V85.1, V85.2, V85.3, V85.4, V85.5, V85.6, V85.7, V85.9, V86, V86.0, V86.1, V86.2, V86.3, V86.4, V86.5, V86.6, V86.7, V86.9, V87, V87.0, V87.1, V87.2, V87.3, V87.4, V87.5, V87.6, V87.7, V87.8, V87.9, V88, V88.0, V88.1, V88.2, V88.3, V88.4, V88.5, V88.6, V88.7, V88.8, V88.9, V89, V89.0, V89.1, V89.2, V89.3, V89.9, V90, V90.0, V90.1, V90.2, V90.3, V90.4, V90.5, V90.6, V90.7, V90.8, V90.9, V91, V91.0, V91.1, V91.2, V91.3, V91.4, V91.5, V91.6, V91.7, V91.8, V91.9, V92, V92.0, V92.1, V92.2, V92.3, V92.4, V92.5, V92.6, V92.7, V92.8, V92.9, V93, V93.0, V93.1, V93.2, V93.3, V93.4, V93.5, V93.6, V93.7, V93.8, V93.9, V94, V94.0, V94.1, V94.2, V94.3, V94.4, V94.5, V94.6, V94.7, V94.8, V94.9, V95, V95.0, V95.1, V95.2, V95.3, V95.4, V95.8, V95.9, V96, V96.0, V96.1, V96.2, V96.8, V96.9, V97, V97.0, V97.1, V97.2, V97.3, V97.8, V98, V99, W00, W01, W02, W03, W04, W05, W06, W07, W08, W09, W10, W11, W12, W13, W14, W15, W16, W17, W18, W19, W20, W21, W22, W23, W24, W25, W26, W27, W28, W29, W30, W31, W32, W33, W34, W341, W342, W343, W344, W349, W35, W36, W37, W38, W39, W40, W41, W42, W43, W44, W45, W46, W49, W50, W51, W52, W53, W54, W55, W56, W57, W58, W59, W60, W64, W65, W66, W67, W68, W69, W70, W73, W74, W75, W76, W77, W78, W79, W80, W81, W83, W84, W85, W86, W87, W88, W89, W90, W91, W92, W93, W94, W99, X00, X01, X02, X03, X04, X05, X06, X08, X09, X10, X11, X12, X13, X14, X15, X16, X17, X18, X19, X20, X21, X22, X23, X231, X232, X233, X239, X24, X25, X26, X27, X28, X29, X30, X31, X32, X33, X34, X35, X36, X37, X38, X39, X40, X43, X44, X46, X47, X48, X50, X51, X52, X53, X54, X57, X58, X59, X59.0, X59.9, Y40, Y40.0, Y40.1, Y40.2, Y40.3, Y40.4, Y40.5, Y40.6, Y40.7, Y40.8, Y40.9, Y41, Y41.0, Y41.1, Y41.2, Y41.3, Y41.4, Y41.5, Y41.8, Y41.9, Y42, Y42.0, Y42.1, Y42.2, Y42.3, Y42.4, Y42.5, Y42.6, Y42.7, Y42.8, Y42.9, Y43, Y43.0, Y43.1, Y43.2, Y43.3, Y43.4, Y43.5, Y43.6, Y43.8, Y43.9, Y44, Y44.0, Y44.1, Y44.2, Y44.3, Y44.4, Y44.5, Y44.6, Y44.7, Y44.9, Y45, Y45.0, Y45.1, Y45.2, Y45.3, Y45.4, Y45.5, Y45.8, Y45.9, Y46, Y46.0, Y46.1, Y46.2, Y46.3, Y46.4, Y46.5, Y46.6, Y46.7, Y46.8, Y47, Y47.0, Y47.1, Y47.2, Y47.3, Y47.4, Y47.5, Y47.8, Y47.9, Y48, Y48.0, Y48.1, Y48.2, Y48.3, Y48.4, Y48.5, Y49, Y49.0, Y49.1, Y49.2, Y49.3, Y49.4, Y49.5, Y49.6, Y49.7, Y49.8, Y49.9, Y50, Y50.0, Y50.1, Y50.2, Y50.8, Y50.9, Y51, Y51.0, Y51.1, Y51.2, Y51.3, Y51.4, Y51.5, Y51.6, Y51.7, Y51.8, Y51.9, Y52, Y52.0, Y52.1, Y52.2, Y52.3, Y52.4, Y52.5, Y52.6, Y52.7, Y52.8, Y52.9, Y53, Y53.0, Y53.1, Y53.2, Y53.3, Y53.4, Y53.5, Y53.6, Y53.7, Y53.8, Y53.9, Y54, Y54.0, Y54.1, Y54.2, Y54.3, Y54.4, Y54.5, Y54.6, Y54.7, Y54.8, Y54.9, Y55, Y55.0, Y55.1, Y55.2, Y55.3, Y55.4, Y55.5, Y55.6, Y55.7, Y56, Y56.0, Y56.1, Y56.2, Y56.3, Y56.4, Y56.5, Y56.6, Y56.7, Y56.8, Y56.9, Y57, Y57.0, Y57.1, Y57.2, Y57.3, Y57.4, Y57.5, Y57.6, Y57.7, Y57.8, Y57.9, Y58, Y58.0, Y58.1, Y58.2, Y58.3, Y58.4, Y58.5, Y58.6, Y58.8, Y58.9, Y59, Y59.0, Y59.1, Y59.2, Y59.3, Y59.8, Y59.9, Y60, Y60.0, Y60.1, Y60.2, Y60.3, Y60.4, Y60.5, Y60.6, Y60.7, Y60.8, Y60.9, Y61, Y61.0, Y61.1, Y61.2, Y61.3, Y61.4, Y61.5, Y61.6, Y61.7, Y61.8, Y61.9, Y62, Y62.0, Y62.1, Y62.2, Y62.3, Y62.4, Y62.5, Y62.6, Y62.8, Y62.9,

	Y63, Y63.0, Y63.1, Y63.2, Y63.3, Y63.4, Y63.5, Y63.6, Y63.8, Y63.9, Y64, Y64.0, Y64.1, Y64.8, Y64.9, Y65, Y65.0, Y65.1, Y65.2, Y65.3, Y65.4, Y65.5, Y65.8, Y66, Y69, Y70, Y70.0, Y70.1, Y70.2, Y70.3, Y70.8, Y71, Y71.0, Y71.1, Y71.2, Y71.3, Y71.8, Y72, Y72.0, Y72.1, Y72.2, Y72.3, Y72.8, Y73, Y73.0, Y73.1, Y73.2, Y73.3, Y73.8, Y74, Y74.0, Y74.1, Y74.2, Y74.3, Y74.8, Y75, Y75.0, Y75.1, Y75.2, Y75.3, Y75.8, Y76, Y76.0, Y76.1, Y76.2, Y76.3, Y76.8, Y77, Y77.0, Y77.1, Y77.2, Y77.3, Y77.8, Y78, Y78.0, Y78.1, Y78.2, Y78.3, Y78.8, Y79, Y79.0, Y79.1, Y79.2, Y79.3, Y79.8, Y80, Y80.0, Y80.1, Y80.2, Y80.3, Y80.8, Y81, Y81.0, Y81.1, Y81.2, Y81.3, Y81.8, Y82, Y82.0, Y82.1, Y82.2, Y82.3, Y82.8, Y83, Y83.0, Y83.1, Y83.2, Y83.3, Y83.4, Y83.5, Y83.6, Y83.8, Y83.9, Y84, Y84.0, Y84.1, Y84.2, Y84.3, Y84.4, Y84.5, Y84.6, Y84.7, Y84.8, Y84.9, Y85, Y85.0, Y85.9, Y86, Y88, Y88.0, Y88.1, Y88.2, Y88.3
**All other causes****	

*Male breast cancer deaths were included in the category “all other cancers” due to small numbers.
**Neonatal deaths (under 28 days of age) are included in the category “all other causes”. For the period 2001 to 2016 they account for 66% (32,484 of 49,144) of deaths in this category below five years of age.

Appendix Table 2: Life expectancy at birth by decile of Index of Multiple Deprivation (IMD), year and sex.

		**Decile of Index of Multiple Deprivation**									
**Year**	**Sex**	**1 Most deprived**	**2**	**3**	**4**	**5**	**6**	**7**	**8**	**9**	**10 Most affluent**
2001	Female	77.2 (77.1, 77.3)	78.6 (78.5, 78.7)	79.4 (79.3, 79.5)	80.4 (80.3, 80.5)	81.0 (80.9, 81.1)	81.3 (81.2, 81.4)	81.8 (81.7, 81.9)	82.0 (81.9, 82.1)	82.5 (82.4, 82.6)	83.3 (83.2, 83.4)
2002	Female	77.2 (77.1, 77.3)	78.5 (78.4, 78.7)	79.4 (79.3, 79.5)	80.4 (80.3, 80.5)	81.0 (80.9, 81.1)	81.4 (81.3, 81.5)	81.8 (81.7, 81.9)	82.1 (82.0, 82.2)	82.5 (82.4, 82.6)	83.3 (83.2, 83.5)
2003	Female	77.0 (76.9, 77.1)	78.4 (78.3, 78.5)	79.3 (79.2, 79.4)	80.3 (80.2, 80.3)	80.9 (80.8, 81.0)	81.3 (81.2, 81.3)	81.8 (81.7, 81.8)	82.0 (81.9, 82.1)	82.5 (82.4, 82.6)	83.3 (83.2, 83.4)
2004	Female	77.6 (77.5, 77.7)	79.0 (78.9, 79.1)	79.9 (79.8, 80.0)	80.8 (80.7, 80.9)	81.5 (81.4, 81.6)	81.9 (81.8, 81.9)	82.3 (82.3, 82.4)	82.6 (82.5, 82.7)	83.1 (83.0, 83.2)	84.0 (83.9, 84.1)
2005	Female	77.7 (77.6, 77.8)	79.1 (79.0, 79.2)	80.0 (79.9, 80.1)	81.0 (80.9, 81.1)	81.6 (81.5, 81.7)	82.0 (81.9, 82.1)	82.5 (82.4, 82.6)	82.8 (82.7, 82.9)	83.3 (83.2, 83.4)	84.2 (84.1, 84.3)
2006	Female	78.0 (77.9, 78.1)	79.4 (79.3, 79.5)	80.5 (80.4, 80.5)	81.4 (81.3, 81.5)	82.0 (81.9, 82.1)	82.4 (82.4, 82.5)	83.0 (82.9, 83.1)	83.2 (83.2, 83.3)	83.8 (83.7, 83.8)	84.7 (84.6, 84.8)
2007	Female	78.1 (78.0, 78.2)	79.5 (79.4, 79.6)	80.6 (80.5, 80.7)	81.5 (81.4, 81.6)	82.2 (82.1, 82.2)	82.6 (82.5, 82.7)	83.1 (83.0, 83.2)	83.4 (83.3, 83.5)	83.9 (83.8, 84.0)	84.9 (84.9, 85.0)
2008	Female	78.0 (77.9, 78.1)	79.5 (79.4, 79.6)	80.6 (80.5, 80.7)	81.5 (81.4, 81.6)	82.2 (82.1, 82.3)	82.6 (82.5, 82.7)	83.2 (83.1, 83.3)	83.4 (83.3, 83.5)	84.0 (83.9, 84.1)	85.0 (84.9, 85.1)
2009	Female	78.7 (78.6, 78.8)	80.1 (80.0, 80.2)	81.2 (81.1, 81.3)	82.2 (82.1, 82.3)	82.8 (82.7, 82.9)	83.2 (83.1, 83.3)	83.8 (83.7, 83.9)	84.1 (84.0, 84.2)	84.7 (84.6, 84.7)	85.7 (85.6, 85.8)
2010	Female	78.8 (78.6, 78.9)	80.2 (80.1, 80.3)	81.3 (81.2, 81.4)	82.2 (82.2, 82.3)	82.8 (82.7, 82.9)	83.3 (83.2, 83.4)	83.9 (83.8, 84.0)	84.2 (84.1, 84.3)	84.7 (84.7, 84.8)	85.9 (85.8, 86.0)
2011	Female	79.1 (79.0, 79.2)	80.5 (80.4, 80.6)	81.6 (81.5, 81.7)	82.6 (82.5, 82.7)	83.2 (83.1, 83.3)	83.7 (83.6, 83.8)	84.3 (84.2, 84.4)	84.6 (84.5, 84.7)	85.2 (85.1, 85.3)	86.3 (86.2, 86.4)
2012	Female	78.9 (78.8, 79.0)	80.4 (80.3, 80.5)	81.5 (81.4, 81.6)	82.4 (82.3, 82.5)	83.0 (82.9, 83.1)	83.6 (83.5, 83.7)	84.1 (84.0, 84.2)	84.4 (84.3, 84.5)	85.1 (85.0, 85.2)	86.2 (86.1, 86.3)
2013	Female	78.9 (78.8, 79.0)	80.4 (80.3, 80.5)	81.6 (81.5, 81.7)	82.4 (82.4, 82.5)	83.1 (83.0, 83.2)	83.6 (83.5, 83.7)	84.2 (84.1, 84.3)	84.5 (84.4, 84.6)	85.1 (85.0, 85.2)	86.3 (86.2, 86.4)
2014	Female	79.2 (79.1, 79.3)	80.7 (80.6, 80.8)	81.9 (81.8, 82.0)	82.8 (82.7, 82.9)	83.5 (83.4, 83.5)	84.0 (83.9, 84.1)	84.6 (84.5, 84.7)	84.9 (84.8, 85.0)	85.5 (85.4, 85.6)	86.7 (86.6, 86.9)
2015	Female	78.7 (78.6, 78.8)	80.2 (80.1, 80.3)	81.4 (81.3, 81.5)	82.3 (82.2, 82.4)	82.9 (82.8, 83.0)	83.5 (83.4, 83.6)	84.1 (84.0, 84.2)	84.5 (84.4, 84.6)	85.1 (85.0, 85.2)	86.3 (86.2, 86.4)
2016	Female	78.8 (78.7, 78.9)	80.4 (80.2, 80.5)	81.6 (81.5, 81.7)	82.5 (82.4, 82.7)	83.2 (83.1, 83.3)	83.8 (83.7, 83.9)	84.4 (84.3, 84.5)	84.8 (84.7, 84.9)	85.4 (85.3, 85.6)	86.7 (86.6, 86.8)
2001	Male	70.8 (70.7, 71.0)	72.8 (72.7, 73.0)	74.2 (74.0, 74.3)	75.5 (75.3, 75.6)	76.3 (76.2, 76.5)	77.1 (77.0, 77.2)	77.8 (77.7, 77.9)	78.2 (78.1, 78.3)	78.7 (78.6, 78.8)	79.8 (79.7, 79.9)
2002	Male	70.9 (70.8, 71.0)	73.0 (72.9, 73.1)	74.2 (74.1, 74.4)	75.6 (75.5, 75.7)	76.5 (76.4, 76.6)	77.2 (77.1, 77.3)	77.9 (77.8, 78.0)	78.3 (78.2, 78.4)	78.9 (78.8, 79.0)	80.0 (79.9, 80.1)
2003	Male	71.0 (70.9, 71.1)	73.1 (73.0, 73.2)	74.4 (74.3, 74.5)	75.8 (75.7, 75.9)	76.7 (76.6, 76.8)	77.4 (77.3, 77.5)	78.0 (77.9, 78.1)	78.5 (78.4, 78.6)	79.0 (78.9, 79.1)	80.2 (80.1, 80.3)
2004	Male	71.5 (71.4, 71.6)	73.6 (73.5, 73.8)	75.0 (74.9, 75.1)	76.3 (76.2, 76.4)	77.2 (77.2, 77.3)	77.9 (77.8, 78.0)	78.6 (78.5, 78.7)	79.1 (79.0, 79.2)	79.6 (79.5, 79.7)	80.7 (80.6, 80.8)
2005	Male	71.8 (71.7, 71.9)	73.9 (73.8, 74.0)	75.3 (75.2, 75.4)	76.6 (76.5, 76.7)	77.5 (77.4, 77.6)	78.2 (78.1, 78.3)	78.8 (78.7, 78.9)	79.4 (79.3, 79.5)	80.0 (79.9, 80.1)	81.1 (81.0, 81.2)
2006	Male	72.0 (71.9, 72.1)	74.2 (74.1, 74.3)	75.6 (75.5, 75.7)	76.9 (76.8, 77.0)	77.9 (77.8, 78.0)	78.6 (78.5, 78.7)	79.3 (79.2, 79.4)	79.7 (79.6, 79.8)	80.3 (80.2, 80.4)	81.5 (81.4, 81.6)
2007	Male	72.3 (72.2, 72.4)	74.4 (74.3, 74.6)	75.9 (75.8, 76.0)	77.2 (77.1, 77.3)	78.2 (78.1, 78.3)	78.9 (78.8, 79.0)	79.5 (79.4, 79.6)	80.1 (80.0, 80.2)	80.7 (80.6, 80.8)	81.8 (81.7, 81.9)
2008	Male	72.4 (72.3, 72.5)	74.6 (74.4, 74.7)	76.1 (76.0, 76.2)	77.4 (77.3, 77.5)	78.3 (78.2, 78.4)	79.1 (79.0, 79.1)	79.7 (79.6, 79.8)	80.2 (80.1, 80.3)	80.9 (80.8, 81.0)	82.0 (81.9, 82.1)
2009	Male	72.9 (72.8, 73.0)	75.1 (74.9, 75.2)	76.5 (76.4, 76.6)	77.8 (77.7, 77.9)	78.8 (78.7, 78.9)	79.5 (79.4, 79.6)	80.2 (80.1, 80.3)	80.7 (80.6, 80.8)	81.3 (81.2, 81.4)	82.4 (82.3, 82.6)
2010	Male	73.3 (73.2, 73.4)	75.4 (75.3, 75.5)	76.8 (76.7, 76.9)	78.1 (78.0, 78.2)	79.0 (78.9, 79.1)	79.8 (79.7, 79.9)	80.4 (80.4, 80.6)	81.0 (80.9, 81.1)	81.6 (81.5, 81.7)	82.7 (82.6, 82.8)
2011	Male	73.7 (73.6, 73.9)	75.9 (75.8, 76.0)	77.3 (77.2, 77.4)	78.6 (78.5, 78.7)	79.5 (79.4, 79.6)	80.2 (80.1, 80.3)	80.9 (80.8, 81.0)	81.4 (81.3, 81.5)	82.0 (81.9, 82.1)	83.2 (83.1, 83.3)
2012	Male	73.9 (73.8, 74.0)	76.0 (75.9, 76.1)	77.4 (77.3, 77.5)	78.7 (78.6, 78.8)	79.5 (79.4, 79.6)	80.3 (80.2, 80.3)	81.0 (80.9, 81.0)	81.5 (81.4, 81.6)	82.1 (82.0, 82.2)	83.3 (83.2, 83.4)
2013	Male	73.9 (73.8, 74.0)	76.0 (75.9, 76.1)	77.4 (77.3, 77.5)	78.7 (78.6, 78.8)	79.6 (79.5, 79.7)	80.3 (80.2, 80.4)	81.0 (80.9, 81.1)	81.5 (81.4, 81.6)	82.1 (82.0, 82.2)	83.3 (83.2, 83.4)
2014	Male	74.1 (74.0, 74.2)	76.3 (76.2, 76.4)	77.7 (77.6, 77.8)	79.0 (78.9, 79.1)	79.9 (79.8, 80.0)	80.7 (80.6, 80.8)	81.3 (81.2, 81.4)	81.8 (81.7, 81.9)	82.4 (82.3, 82.5)	83.7 (83.6, 83.8)

2015	Male	73.9<br>(73.8, 74.0)	76.0<br>(75.9, 76.1)	77.4<br>(77.3, 77.5)	78.7<br>(78.6, 78.8)	79.6<br>(79.5, 79.7)	80.4<br>(80.3, 80.5)	81.1<br>(81.0, 81.2)	81.6<br>(81.5, 81.7)	82.2<br>(82.1, 82.3)	83.4<br>(83.3, 83.5)
2016	Male	74.0<br>(73.9, 74.1)	76.2<br>(76.0, 76.3)	77.6<br>(77.4, 77.7)	78.9<br>(78.8, 79.0)	79.9<br>(79.8, 80.0)	80.6<br>(80.5, 80.7)	81.3<br>(81.2, 81.4)	81.8<br>(81.7, 82.0)	82.4<br>(82.3, 82.5)	83.8<br>(83.6, 83.9)

Appendix Table 3: Age standardised death rates (per 100,000 people) in 2016 by cause of death, decile of Index of Multiple Deprivation (IMD), and sex.

		Decile of Index of Multiple Deprivation										Ratio of ASDRs, decile 1 to decile 10
		1 Most deprived	2	3	4	5	6	7	8	9	10 Most affluent	
**Cause**	**Sex**	**Age standardised death rates (per 100,000 people)**										
Respiratory diseases	Female	180.2	149.3	130.3	116.3	103.8	95.9	89.8	87.6	77.4	68.4	2.6
Respiratory diseases	Male	238.5	209.2	182.3	158.3	143.2	132.5	125.2	117.5	110.5	95	2.5
Ischaemic heart disease	Female	99.9	87.4	77.7	70.3	65	60.6	56.8	54.6	49.7	43.6	2.3
Ischaemic heart disease	Male	216.8	185.5	170.7	152.4	138.6	129.9	121.7	115.4	107.7	96.3	2.3
Stroke	Female	65	60.7	59.1	55.3	54.2	53.8	51.1	49.1	47.5	42.6	1.5
Stroke	Male	74.6	71.9	64.9	60.2	57.6	55.9	53.4	52.1	49.3	43.8	1.7
Other circulatory diseases	Female	84.7	79.4	77.1	71.8	71	69.7	64	60.2	56.6	51.3	1.7
Other circulatory diseases	Male	103.5	95.7	93	84.5	80.2	76.9	72.8	71.3	69	61	1.7
Diabetes	Female	13.8	11.9	10.2	9.2	8.5	7.2	6.9	6.5	5.6	5.2	2.7
Diabetes	Male	18.8	17.1	15	13	11.5	10.1	10.2	9.3	8	6.9	2.7
Cancer, liver	Female	8.9	7.6	6.2	6.2	5.9	5.4	5.4	5	4.9	4.4	2.0
Cancer, liver	Male	16.5	13.6	13.1	12.3	10.1	10.2	9.5	9	8.7	7.7	2.1
Cancer, digestive organs	Female	62	57.4	54.6	53.2	50.7	49	47.6	46.2	46.6	44	1.4
Cancer, digestive organs	Male	105.6	97.9	90.7	85.8	81.1	79.4	79.2	74.6	73.7	67.5	1.6
Cancer, haematological	Female	16.5	16.8	16.7	16	15.2	15.5	15.1	15	15.2	14.5	1.1
Cancer, haematological	Male	26.9	26.3	25.9	27.4	25.6	25	26.2	24.9	25.4	24.4	1.1
Cancer, lung	Female	82.7	68.1	57.2	49.3	42.1	38.9	34.4	32.2	29.2	24.7	3.3
Cancer, lung	Male	118.8	97.6	83.1	72.1	62.6	56.4	51.7	47.9	43.5	37.2	3.2
Cancer, prostate	Male	43.6	44.5	43.5	44.2	43	45	43.6	42.4	42.8	41	1.1
Cancer, breast	Female	33.2	33.6	31.4	33.1	31.6	31.1	31.7	31.8	31.5	29.3	1.1
Cancer, other	Female	81.5	77.1	74.3	70.6	69.7	67	65.3	64.4	62.2	58.1	1.4
Cancer, other	Male	103.1	95.6	88.3	83.7	83.4	79.4	79.2	76.2	75.7	70.4	1.5
Alzheimer's disease and other dementias	Female	136.7	144.7	129.5	120.3	118.2	117.6	110.6	107.7	100.5	87	1.6
Alzheimer's disease and other dementias	Male	123.2	123.4	109.5	98	95.2	93.2	86.7	83.9	82.2	70.5	1.7
Intentional injuries	Female	4.6	3.5	3.6	3.5	3	3.8	3.2	3.2	3.2	3	1.5
Intentional injuries	Male	16.3	13.3	12.5	12	10.7	11.3	10.5	9.6	8.5	8.5	1.9
Unintentional injuries	Female	21.9	20.7	18.1	16.7	16.7	15.9	15.1	14.6	13.6	12.9	1.7
Unintentional injuries	Male	39.5	33.6	30.3	29.7	27.8	26.5	25	23.5	23.1	20.3	1.9
All other causes	Female	183.5	163	147.1	137.5	132.2	124.1	119.6	115.4	110	95.2	1.9
All other causes	Male	241.4	203.8	182.4	161.7	148.6	143	133	128.6	121.1	105.7	2.3
All causes	Female	1075.1	981.2	892.9	829.4	787.9	755.6	716.8	693.5	653.7	584.2	1.8
All causes	Male	1487.1	1328.9	1205.3	1095.3	1019.5	974.7	927.9	886	849.3	756.1	2.0
